# Mebendazole and temozolomide in patients with newly diagnosed high-grade gliomas: results of a phase 1 clinical trial

**DOI:** 10.1093/noajnl/vdaa154

**Published:** 2020-11-12

**Authors:** Gary L Gallia, Matthias Holdhoff, Henry Brem, Avadhut D Joshi, Christine L Hann, Ren-Yuan Bai, Verena Staedtke, Jaishri O Blakeley, Soma Sengupta, T Che Jarrell, Jessica Wollett, Kelly Szajna, Nicole Helie, Austin K Mattox, Xiaobu Ye, Michelle A Rudek, Gregory J Riggins

**Affiliations:** 1 Sidney Kimmel Comprehensive Cancer Center at Johns Hopkins University, Department of Neurosurgery, Johns Hopkins University School of Medicine, Baltimore, Maryland, USA; 2 Department of Neurology, Johns Hopkins University School of Medicine, Baltimore, Maryland, USA; 3 Department of Oncology, Johns Hopkins University School of Medicine, Baltimore, Maryland, USA; 4 Department of Medicine, Division of Clinical Pharmacology, Johns Hopkins University School of Medicine, Baltimore, Maryland, USA; 5 Milestone Regulatory Experts, Gulfport, Florida, USA; 6 Department of Neurology and Rehabilitation Medicine, University of Cincinnati College of Medicine, Cincinnati, Ohio, USA

**Keywords:** dose escalation, glioblastoma, malignant glioma, mebendazole, phase 1 clinical trial

## Abstract

**Background:**

Mebendazole is an anthelmintic drug introduced for human use in 1971 that extends survival in preclinical models of glioblastoma and other brain cancers.

**Methods:**

A single-center dose-escalation and safety study of mebendazole in 24 patients with newly diagnosed high-grade gliomas in combination with temozolomide was conducted. Patients received mebendazole in combination with adjuvant temozolomide after completing concurrent radiation plus temozolomide. Dose-escalation levels were 25, 50, 100, and 200 mg/kg/day of oral mebendazole. A total of 15 patients were enrolled at the highest dose studied of 200 mg/kg/day. Trough plasma levels of mebendazole were measured at 4, 8, and 16 weeks.

**Results:**

Twenty-four patients (18 glioblastoma and 6 anaplastic glioma) were enrolled with a median age of 49.8 years. Four patients (at 200 mg/kg) developed elevated grade 3 alanine aminotransferase (ALT) and/or aspartate transaminase (AST) after 1 month, which reversed with lower dosing or discontinuation. Plasma levels of mebendazole were variable but generally increased with dose. Kaplan–Meier analysis showed a 21-month median overall survival with 41.7% of patients alive at 2 years and 25% at 3 and 4 years. Median progression-free survival (PFS) from the date of diagnosis for 17 patients taking more than 1 month of mebendazole was 13.1 months (95% confidence interval [CI]: 8.8–14.6 months) but for 7 patients who received less than 1 month of mebendazole PFS was 9.2 months (95% CI: 5.8–13.0 months).

**Conclusion:**

Mebendazole at doses up to 200 mg/kg demonstrated long-term safety and acceptable toxicity. Further studies are needed to determine mebendazole’s efficacy in patients with malignant glioma.

**Key Points**

1. High-dose oral mebendazole plus standard monthly temozolomide is safe in patients with newly diagnosed high-grade gliomas in the adjuvant setting.
2. The most common side effect was reversible elevation of liver enzymes.
3. Further clinical evaluation of mebendazole in patients with high-grade gliomas is warranted to better evaluate a potential benefit from this regimen.

**Importance of the Study**

Preclinical studies suggest that the antiparasitic drug, mebendazole, has anticancer activity in several cancer types, including glioblastoma and other brain cancers. Here, we establish safety and dosing of high-dose mebendazole in combination with temozolomide during a 6-year observation period. The lack of toxicity and encouraging (but not statistically significant) survival supports further evaluation in a phase 2 study.

Mebendazole is a successful anthelminthic drug introduced in 1971 that is available worldwide either by prescription or, as seen more commonly in tropical countries, sold over the counter.^1^ Its antiparasitic mechanism has been attributed to binding of mebendazole to tubulin monomers in the gut of the worm, preventing tubulin polymerization and subsequently limiting absorption of nutrients in the gut of the parasite.^1–4^

Mebendazole has shown activity against various preclinical cancer models that include adrenocortical carcinoma, colon cancer, gliomas, lung cancer, breast cancer, and others.^3–12^ There is a case report of long-term control with mebendazole in a patient with advanced adrenocortical carcinoma and there is an effort that includes clinical trials to use mebendazole for colon cancer.^7,10,13,14^ While mebendazole’s antiparasitic mechanism was first recognized as being associated with anticancer growth, mebendazole has also been implicated as a multityrosine kinase inhibitor with several anticancer targets that include VEGFR2, BRAF and ERK.^6,7,11,15^

Research reports on the anticancer activity of this repurposed drug have garnered the attention of patients and patient families seeking better therapies, but there are no reports of clinical trials to document its safety at the high doses in cancer patients or in combination with cancer therapy. This information on safety, toxicity, and dosing is necessary to determine if efficacy trials are warranted. Based on the encouraging preclinical activity of mebendazole in preclinical cancer models, clinical trials in colon (NCT03925662) and brain cancers (NCT02644291) are underway (http://www.ClinicalTrials.gov).

Due to a serendipitous finding of activity of this benzimidazole class of antiparasitic in brain tumors, the subsequent demonstration of activity of mebendazole in 2 different mouse models of glioblastoma,^3^ and mebendazole’s ability to cross the blood–brain barrier,^16^ we initiated and fully enrolled a phase 1 trial using mebendazole for newly diagnosed patients with high-grade glioma.

Here, we report a summary of our safety and survival findings for 24 patients with newly diagnosed high-grade glioma enrolled in a single arm dose-escalation study (NCT01729260). Patients were treated with mebendazole in combination with standard temozolomide after completion of 6 weeks of standard postoperative chemoradiation. The primary goals of the study were to determine maximum tolerated dose (MTD) when using mebendazole in combination with temozolomide and evaluate safety and toxicity at the MTD. Secondary goals were to estimate plasma levels and obtain preliminary estimate of median overall survival (mOS) and median progression-free survival (mPFS).

## Material and Methods

### Mebendazole and IND

At the time of the start of the study, commercial mebendazole was not available for purchase in the United States, despite mebendazole being an FDA-approved anthelmintic. We sought and obtained a research Investigational New Drug Application (IND) from the FDA for a custom noncommercial production of mebendazole polymorph C.^16^ The mebendazole polymorph C used in this study was bound in orange-flavored chewable tablets and provided in 500 mg doses. These tablets were dispensed by the Investigational Drug Services pharmacy at Johns Hopkins Cancer Center. Tablets were authorized for experimental use only for approved clinical trials under the IND.

### Eligibility Criteria

Patients aged 18 years or older with a histologically confirmed newly diagnosed malignant glioma (WHO Grade III or IV) were candidates for this study. Eligibility criteria included a KPS ≥ 60%, life expectancy greater than 12 weeks, adequate organ and marrow function (leukocytes ≥ 3,000/mcL, absolute neutrophil count ≥ 1,500/mcL, platelets ≥ 100,000/mcL, AST/ALT ≤ 2.5 × upper limit of normal (ULN), total bilirubin ≤ 1.5 × ULN, and creatinine ≤ 1.5 × ULN or creatinine clearance ≥ 60 mL/min/1.73 m^2^ for patients with creatinine ≥ 1.5 × ULN), and completion of > 80% of the prescribed radiation therapy and concurrent temozolomide according to the Stupp regimen^17^ without grade 3 or 4 hematologic toxicity. Additional inclusion criteria included ability to understand and willingness to sign a written informed consent document, ability to comply with treatment plan, study procedures, and follow-up examinations, and ability to swallow pills and keep medication record. The protocol allowed for previous implantation of polifeprosan 20 with carmustine wafer during tumor resection. Exclusion criteria included prior therapy other than standard chemoradiation according to Stupp^17^ and carmustine polymer wafer implant, concurrent investigational agents while on study, known allergy or severe side effect to mebendazole or benzimidazole, taking metronidazole,^18^ taking any benzimidazole in past 3 months, taking any anticonvulsant that interferes with cytochrome P450 pathway (phenytoin, phenobarbital or carbamazepine),^19^ pregnancy, and uncontrolled intercurrent illness.

### Clinical Trial Design

This was single-institution, open-label, nonrandomized phase 1 trial to define the MTD of mebendazole in combination with temozolomide given after RT and temozolomide among patients with newly diagnosed malignant gliomas using a standard 3 + 3 design. Oral mebendazole was started concurrently with standard adjuvant temozolomide.^17^ In brief, the sequence of therapy was surgery, temozolomide plus radiation therapy following the Stupp protocol, a recuperation period targeted at one month, concurrent temozolomide plus oral mebendazole for 6–12 months, and mebendazole monotherapy until documented progression or patient withdrawing from the study. Mebendazole dose levels were 25, 50, 100, and 200 mg/kg/day of chewable mebendazole 500 mg tablets in 3 divided doses with meals. Dosing was rounded either up or down to the nearest 500 mg increment and no weight adjustment was made for obese patients. An expansion cohort to a total of 15 patients at the MTD, or at 200 mg/kg/day if an MTD is not determined, was planned as part of this study.

### Patient Evaluations

Baseline evaluations included brain MRI obtained 1 month postcompletion of chemoradiation, medical history and physical examination, complete blood count (CBC) with differential, comprehensive metabolic panel (CMP), pregnancy test (where appropriate), KPS, and pain assessments. After starting mebendazole, study visits coincided with standard of care visits every 4 weeks and included interval medical history, physical exam, CBC with differential and CMP, KPS, pain assessment, review of mebendazole treatment administration records, and documentation of any adverse events (AEs) or serious AEs. Brain MRI scans were performed at the end of every other cycle starting at the end of adjuvant chemotherapy cycle 2 (week 8). Participants who discontinued temozolomide but were still receiving mebendazole were seen every 4 weeks for assessment. There were no limits on the number of mebendazole cycles. Mebendazole treatment continued until tumor progression, toxicity, failure to take ≥ 80% of mebendazole since study day 1, or patient withdrawal from the study. Radiographic response was assessed by Response Assessment in Neuro-Oncology (RANO) criteria.^20^ All patients were followed during mebendazole treatment and after discontinuation of mebendazole treatment until death.

### Dose Escalation

A standard 3 + 3 design was used for dose escalation. Three patients were planned to be treated at each dose level with an expansion to a total of 15 patients at the MTD or the highest dose level of 200 mg/kg daily, whichever was lower. Determination of MTD was based on the assessment of dose-limiting toxicities (DLT) of mebendazole and was defined as the dose with ≤ 33% DLT. The safety evaluation period for dose escalation was 28 days. The MTD was defined as the mebendazole dose with 0 or 1 of 6 patients having a DLT, or at one dose below the dose level at which 2 or more of 6 patients have a DLT. Every 28 day cycle the patient had a blood count and serum chemistry performed and evaluated by the physician. AEs were graded based on the Common Terminology Criteria for Adverse Events (CTCAE) version 4 and reported to the FDA and local IRB. DLT was defined as any dose that lead to any CTCAE version 4.0 grade ≥ 3 nonhematologic or grade ≥ 4 hematologic toxicity. The study was registered at clinical trials.gov (NCT01729260), and participants signed a written informed consent approved by the Institutional Review Board.

### Mebendazole Pharmacokinetics

Plasma samples were collected at trough prior to mebendazole administration (minimum concentration [*C*_min_]) and at 4, 8, and 16 weeks on study. The concentrations of mebendazole and its 2 metabolites, 2-amino-5-benzoyl-benzimidazole and rac dihydro mebendazole, were determined using a validated liquid chromatography–mass spectrometry assay, over the range of 5–500 ng/mL (mebendazole) and 1–500 ng/mL (metabolites) with dilutions of up to 1:10 (v:v) as previously described.^16^ There is no reported evidence that any of the metabolites show activity to cancer, but were included in the study. Samples that were not determined to be pretreatment were not utilized in the assessment of steady-state concentrations. Steady-state plasma trough concentrations were calculated as the average of pretreatment concentrations at 4, 8, and 16 weeks. The ratio of the metabolite to parent drug was calculated on the average steady-state concentrations.

### Statistical Methods

The study was designed to define a MTD of mebendazole in combination with temozolomide per standard-of-care. A standard 3 + 3 design was used for dose escalation with a cohort expansion at the MTD or the highest safe dose to a total of 15 patients. The targeted DLT rate was ≤ 33%. The safety evaluation period for the dose escalation was 28 days.

Descriptive statistics were used to summarize patient characteristics and toxicity data. Survival probability was estimated using the method of Kaplan–Meier. The pharmacokinetic parameters that were compared were differences in dose normalized exposure and metabolite to mebendazole ratios as a function of dose level and evaluated for statistical significance using a Kruskal–Wallis analysis of variance by ranks with post hoc analysis using an All Pairs Tukey–Kramer test. Mann–Whitney *U*-tests were used to assess correlations between mebendazole and metabolite exposure and response or toxicity. The a priori level of significance was set at *P* < .05.

## Results

### Patient and Trial Characteristics

A total of 24 patients with newly diagnosed high-grade glioma were consented and enrolled. Patients and disease baseline characteristics are summarized in Table 1. Of the 24 patients, 2 had IDH-mutant tumors, 18 had IDH wild type, and 4 had tumors of unknown IDH status. Patients completed surgery, radiation plus temozolomide and had a recuperation period averaging 36 ± 7.2 days. Patients started the prescribed dose of mebendazole on the first day of what would normally be temozolomide only, that continued for 6–12 months of temozolomide at 75 mg/m^2^. Patients continued daily mebendazole between cycles of temozolomide and after the end of temozolomide therapy and were eligible to receive mebendazole until documented disease progression, toxicity, or patient withdraw from the study.

**Table 1.** Baseline Characteristics of Study Patients (*n* = 24)

Age (years):	
Median (range)	49.8 (27.8–67.5)
<50	12 (50%)
50–68	12 (50%)
Gender: no. (%)	
Male	15 (63)
Race: no. (%)	
White	24 (100)
KPS: no. (%)	
100	4 (17)
90	12 (50)
80	6 (25)
60	1 (4)
Missing	1 (4)
Surgical procedure: no. (%)	
Gross total resection	4 (17)
Near total resection	2 (8)
Partial resection	8 (33)
Open biopsy	1 (4)
Resection, not specified	9 (38)
Diagnosis: no. (%)	
Glioblastoma	18 (75)
Anaplastic astrocytoma	5 (21)
Anaplastic infiltrating glioma	1 (4)
MGMT: no. (%)	
Methylated:	5 (21)
Unmethylated:	13 (54)
Unknown:	6 (25)
IDH1: no. (%)	
Mutated (AA)	2 (8)
Wildtype	18 (75)
Unknown (grade IV)	4 (17)

### Safety

There were no DLTs, defined as grade 4 or above AEs for hematologic events or grade 3 or above for events other than hematologic, observed during the first month of mebendazole plus temozolomide. This first cycle lack of actionable AEs resulted in dose escalation to the highest planned dose level of 200 mg/kg/day. During the subsequent observation period from months 2 to 5, delayed toxicity of elevated liver enzymes at grade 3 attributable to mebendazole was observed at the highest dose level of 200 mg/kg/day in 4 patients (16.7%) (Table 2). Elevated AST was observed in 2 patients and elevated ALT was observed in all 4. The ALT and AST elevations were reversible to normal levels; in 3 cases dose reduction to 100 mg/kg and in 1 case with mebendazole discontinuation. The only additional grade 3 events attributed to mebendazole beyond month 5 were also elevated ALT or AST. These additional occurrences were in 2 of the same patients that had elevated liver enzymes during months 2 to 5, and one patient had four additional AEs and had to come off trial, at which time her enzymes returned to normal.

**Table 2.** Dose-Limiting Toxicity and Adverse Events Requiring Action Through Cycle 5

MBZ Dose (mg/kg/day)	No. of Patients	DLT During First Cycle	No. of Patients With DLT Cycles 2–5	Type of DLT
25	3	None	None	
50	3	None	None	
100	3	None	None	
200	15	None	4	6 DLTs: 4 ALT, 2 AST (all grade 3)

ALT, alanine aminotransferase; AST, aspartate transaminase; DLT, dose-limiting toxicity.

During the entire trial, no severe AEs (those requiring hospitalization or resulting in death) were observed that were attributable to mebendazole. The trial is ongoing with 2 patients still taking the investigational mebendazole now over 5 or 6 years on treatment, 1 patient with an IDH-mutant anaplastic astrocytoma and 1 with an unknown IDH status glioblastoma.

### Pharmacokinetics

Seventeen of 24 patients had sufficient plasma samples to determine steady-state plasma concentrations of mebendazole and its metabolites. The mean plasma mebendazole, 2-amino-5-benzoyl-benzimidazole, and rac dihydro mebendazole trough concentrations (*C*_min_) and ratio of metabolite to parent drug by dose level are presented in Table 3. There was a statistically significant difference in dose-normalized exposure for mebendazole (*P* = .04), 2-amino-5-benzoyl-benzimidazole (*P* = .008), and rac dihydro mebendazole (*P* = .02) with the 200 mg/kg having the lowest dose-normalized exposure. The metabolite to mebendazole ratios did not differ by dose level. The mebendazole metabolites have not been studied for anticancer activity. There were no statistically significant correlations between response or the worst grade of toxicity and mebendazole or metabolite exposure (*P* > .05).

**Table 3.** Steady-State Plasma Concentrations of Mebendazole and Metabolites; 2-amino-5-benzoyl-benzimidazole and rac dihydro mebendazole

Dose Level (mg/kg)	*n*	Mebendazole (ng/mL)*	2-amino-5-benzoyl-benzimidazole (ng/mL)	2-amino-5-benzoyl-benzimidazole: mebendazole ratio	rac dihydro mebendazole (ng/mL)	rac dihydro mebendazole: mebendazole ratio
25	2	44.9, 79.9	129.3, 157.0	1.6, 3.5	281.3, 294.0	3.5, 6.5
50	3	192.2 ± 131.4	299.0 ± 47.4	1.9 ± 0.9	747.3 ± 411.1	4.1 ± 0.5
100	2	225.0, 480.0	270.5, 383.0	0.6, 1.7	939.0, 2,035.0	4.2, 4.2
200	10	261.0 ± 126.4	351.5 ± 79.4	1.7 ± 0.8	1,242.9 ± 600.7	4.9 ± 1.1

*When only 2 patients were measured both values are reported separated by a comma. If 3 or more patients were measured than the average ± the standard deviation is given.

### Survival

**Figure 1.**
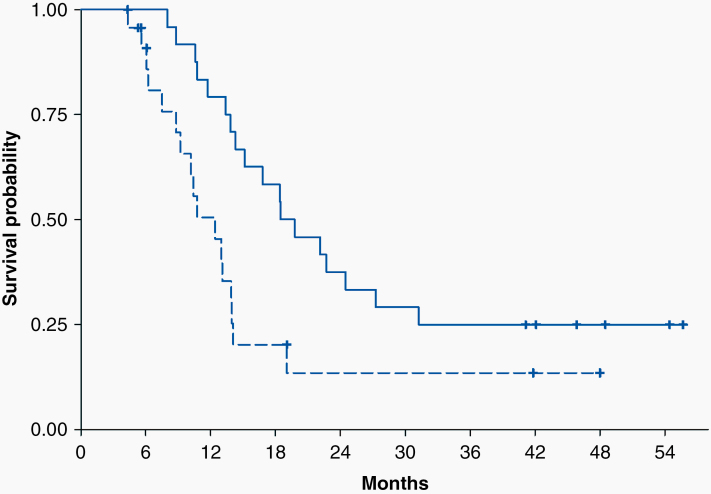
Kaplan–Meier Analysis of all 24 study patients (18 glioblastomas, 6 anaplastic gliomas) shows 21-month median overall survival with 41.7% of patients alive at 2 years and 25% at 3 and 4 years. Hash marks are censured observations. The solid line is overall survival, and the dotted line is progression free survival.

Mean overall survival from date of diagnosis for all 24 patients was 21.0 months by Kaplan–Meier analysis (95% confidence interval [CI]: 14.3–31.2) (Figure 1). This same analysis showed that 41.7% of patients were alive at 2 years and 25% at 3 and 4 years. The overall survival from the date of starting mebendazole plus temozolomide TMZ was 17.0 months (95% CI: 9.9–27.8). There were 6 survivors after 4 years of therapy, 2 with known IDH-mutant glioma, one with IDH-wildtype glioma, and 3 in whom the IDH status was unknown. Of these 6 patients, 4 patients had tumors with methylated O^6^-methylguanine-DNA methyltransferase (MGMT), 1 with unmethylated, and 1 with MGMT methylation status unknown. Two long-term survivors had gross-total resections, 1 had a near gross-total resection, 2 had had partial resections, and 1 was not reported. There was no correlation between dosing or number of cycles of the experimental drug and survival. Two patients are still on trial at the time of this writing with over 5 years of mebendazole oral therapy.

Progression-free survival (PFS) from the date of diagnosis for all 24 patients was 13.0 months (95% CI: 9.2–14.1). PFS for 17 patients receiving more than 1 cycle of mebendazole was 13.1 (95% CI: 8.8–14.6) months, while for 7 patients receiving less than 1 cycle PFS was 9.2 months (95% CI: 5.8–13.0).

## Discussion

Mebendazole, a drug originally developed in the 1970s for anthelmintic use, has demonstrated anticancer efficacy and its mechanism investigated in numerous preclinical studies,^3,4,7–9,11,14,16,21–26^ but has yet to be studied extensively in human clinical trials. In this phase 1 trial of patients with newly diagnosed high-grade gliomas, we evaluated mebendazole dosing of up to 200 mg/kg/day in combination with temozolomide, report on its safety, acceptable toxicity, and plasma concentrations. Most notable for this study is that there were no severe AEs attributed to relatively high dosing of the investigational drug. There are several ongoing trials using mebendazole as an anticancer agent for colon and brain cancers, and the first steps reported here are potentially useful for optimizing the design of future efficacy trials.

Mebendazole is metabolized primarily by the liver, and the only delayed toxicity observed was elevated ALT and AST, a potential sign of liver toxicity, although there was no elevated bilirubin observed in this study. Elevated liver enzymes were observed primarily in conjunction with temozolomide but did not exceed grade 3 and occurred at the highest dose level. Neutropenia has been reported at rates of about 5% with comparable high oral dosing in patients with hydatid disease from *Echinococcus granulosus*,^27–29^ and in this study, there were 3 grade 3 events, one of which occurred with mebendazole only. However, as none of these hematologic events exceeded grade 3, there was no action required to lower dosing. Serious AEs were absent with this trial due to the safety of the drug, and safety monitoring with monthly blood counts and serum chemistry. For any future use of mebendazole at these doses for more than 1 month, we recommend this simple and inexpensive safety measure.

While finding a lack of severe AEs and limited toxicity was not surprising given the history of this drug’s safe use, it was necessary to further document the drug’s safety for the high-grade glioma patient population, given a much higher dosage and duration and the lack of a known toxicity profile when combining mebendazole with temozolomide chemotherapy. This phase 1 clinical study supports that oral mebendazole can be used safely in high doses in combination with temozolomide.

We proposed and achieved dose escalation up to the highest published long-term dose of 200 mg/kg/day of oral mebendazole. This dosing is based on a previous study of 37 children who were treated for hydatid disease with 100–200 mg/kg/day of mebendazole without serious side effects.^30^ Mebendazole exposure was highly variable (48–68%) with a potential decrease in dose-normalized exposure at the 200 mg/kg/day. Delayed toxicity was only observed at the highest dose of 200 mg/kg/day, which may be due to the higher dose, the greater number of patients tested, or both. Although the 200 mg/kg/day dose level did encounter late toxicity, it is not considered the MTD as none of the AEs were encountered during the initial dose-escalation period. However, there are considerations as to why levels less than 200 mg/kg/day might be preferable. Some patients did complain about the pill burden at the highest dose which often amounted to 8 or more tablets per meal. There was a possible decrease of plasma levels at 200 mg/kg/day, delayed toxicity, and potential challenges with compliance that all suggest a reasonable dose for future trials would be in the 75–100 mg/kg/day range or a formulation with greater bioavailability that requires fewer pills per dose.

There are different polymorphs of mebendazole, and we used mebendazole polymorph C, which has a higher gastrointestinal absorption than polymorph A.^16^ Mebendazole is brain penetrant with a brain to plasma ratio of approximately 75% in mice.^16^ However, in mice, plasma levels observed with effective doses of mebendazole at 6 h were in excess of 1,000 ng/mL. Further studies would be required to see if these levels could be safely achieved in humans, including with a prodrug of mebendazole that can potentially reach higher plasma levels.^31^

The known molecular mechanism of mebendazole for antiparasitic use is the binding of tubulin to prevent is polymerization, an anticancer mechanism observed in animal models of glioblastoma.^3^ Mebendazole may also target kinases. Mebendazole has been reported as a kinase inhibitor of TRAF2- and NCK-interacting kinase (TNIK),^32^ vascular endothelial growth factor receptor 2 (VEGFR2),^11^ dual specificity tyrosine-phosphorylation-regulated kinase 1B (DYRK1B),^33^ and both BRAF wild type and BRAFV600E.^6^ Although targeted kinase inhibition has not demonstrated success in clinical trials for glioblastoma, mebendazole simultaneously combines multiple molecular targets in a single brain penetrant and well tolerated oral drug.

In this safety trial, as with others, survival determinations are preliminary owing to the small study size, heterogenous population (eg, inclusion of both WHO grade III and IV tumors) and sample bias. While a 21-month median survival and 4-year survival of 25% may sound promising, it may be difficult to reproduce this in a larger trial that focuses only on grade IV glioblastomas. However, this study and animal studies^16^ support that mebendazole given at these doses does not interfere the standard of care temozolomide, is safe in combination and may enhance survival.

There is considerable public interest in new therapies for glioblastoma, and mebendazole is widely available for antiparasitic use. We do not recommend its use outside the care of a qualified oncologist for several reasons. Use of mebendazole has not been optimized for oncology and efficacy has not been established in a randomized clinical trial. There are side effects, most notably elevated liver enzymes and low blood cell counts that if not detected and dosages adjusted or therapy stopped, could be potentially harmful. Additionally, the bioavailability of mebendazole preparations differs substantially rendering certain formulations likely unsuitable for oncology patients.^16^ Furthermore, mebendazole should not eliminate the proven beneficial standard of care, and in this study, it was added to standard of care.

A key component in the standard of care for glioblastoma is radiation, and recent data indicate that mebendazole plus radiation provides a survival benefit beyond either alone in preclinical models of triple negative breast cancer and intracranial malignant meningioma.^5,34^ Because mebendazole appears to have low toxicity when used in combination with temozolomide, it opens the possibility for further testing in combination with of the standard of care including radiation and perhaps eventually with promising experimental therapies. The combination of mebendazole plus other therapies for intracranial malignancies may increase efficacy, while not substantially increasing therapy induced toxicity. While this preliminary study had too few patients to demonstrate efficacy, we conclude that mebendazole has sufficient safety, plasma levels, and lack of toxicity to proceed to randomized phase II trials.

## Notes

**Conflict of interest statement.** G.J.R. is a founder of and holds equity in Benizole Therapeutics, PBC. The results of the study discussed in this publication could affect the value of Benizole. This arrangement has been reviewed and approved by the Johns Hopkins University in accordance with its conflict of interest policies. H.B. receives research funding from Acuity Bio Corp* and is a consultant for AsclepiX Therapeutics, StemGen, Nurami Medical*, Galen Robotics, Inc.* and Camden Partners* (*includes equity or options).

## Notes

**Authorship Statement.** Conception, design, and development of methodology: G.L.G., H.B., C.L.H., X.Y., G.J.R. Acquisition, analysis, and interpretation of data: X.Y., G.L.G., G.J.R, A.K.M., K.S., N.H. Writing and revision of manuscript: C.L.H., G.L.G., G.J.R., A.K.M., S.S. Clinical study supervision and IRB: G.L.G., J.W. Federal Regulatory Affairs: T.C.J., G.J.R. Statistics: X.Y. Pharmacokinetics: M.A.R. Drug supply and quality control. A.D.J., R.-Y.B., G.J.R. Patient enrollment and care: G.L.G., M.H., V.S., J.O.B., H.B. Study sponsor responsibilities and IND: G.J.R.
